# It's Not the Flu: Popular Perceptions of the Impact of COVID-19 in the U.S.

**DOI:** 10.3389/fpsyg.2021.668518

**Published:** 2021-05-07

**Authors:** Laura Niemi, Kevin M. Kniffin, John M. Doris

**Affiliations:** ^1^Department of Psychology, Cornell University, Ithaca, NY, United States; ^2^Charles H. Dyson School of Applied Economics and Management, SC Johnson College of Business, Cornell University, Ithaca, NY, United States; ^3^Sage School of Philosophy, Cornell University, Ithaca, NY, United States

**Keywords:** COVID-19, pandemics, prosocial behavior, moral psychology, moral values, consumer psychology

## Abstract

Messaging from U.S. authorities about COVID-19 has been widely divergent. This research aims to clarify popular perceptions of the COVID-19 threat and its effects on victims. In four studies with over 4,100 U.S. participants, we consistently found that people perceive the threat of COVID-19 to be substantially greater than that of several other causes of death to which it has recently been compared, including the seasonal flu and automobile accidents. Participants were less willing to help COVID-19 victims, who they considered riskier to help, more contaminated, and more responsible for their condition. Additionally, politics and demographic factors predicted attitudes about victims of COVID-19 above and beyond moral values; whereas attitudes about the other kinds of victims were primarily predicted by moral values. The results indicate that people perceive COVID-19 as an exceptionally severe disease threat, and despite prosocial inclinations, do not feel safe offering assistance to COVID-19 sufferers. This research has urgent applied significance: the findings are relevant to public health efforts and related marketing campaigns working to address extended damage to society and the economy from the pandemic. In particular, efforts to educate the public about the health impacts of COVID-19, encourage compliance with testing protocols and contact tracing, and support safe, prosocial decision-making and risk assessment, will all benefit from awareness of these findings. The results also suggest approaches, such as engaging people's stable values rather than their politicized perspectives on COVID-19, that may reduce stigma and promote cooperation in response to pandemic threats.

## Introduction

As the novel coronavirus (COVID-19) emerged in late 2019, and the ensuing pandemic claimed tens of thousands of American lives in the Spring and Summer of 2020, U.S. leadership delivered conflicting messages to the public. Famously, President Trump repeatedly expressed a lack of concern, equating COVID-19 with the seasonal flu and deaths from automobile accidents. For example, on March 9, 2020, when the U.S. stock market plummeted over fears of the spread of the coronavirus, he tweeted, “…last year 37,000 Americans died from the common Flu. It averages between 27,000 and 70,000 per year. Nothing is shut down, life and the economy go on.” In stark contrast, public health officials contested such sanguine assessments. For example, pandemic expert Irwin Redlener, director of the National Center for Disaster Preparedness, declared, “It's not responsible of governors to rush into a return to business as usual, even if it's relatively slow,” he said. “This is a serious risk. We're playing with fire” (Bredderman and Messer, 2020).

Unsurprisingly, given this conflicting messaging from authorities, Americans' response to the pandemic was highly variable. While many people defied, and even protested, requirements for mask wearing, social distancing, and economic shutdowns, many others viewed the pandemic with considerable alarm, even taking precautions before they were required (Bump, 2020; Burnett, 2020; Dave et al., 2020; Malone and Bourassa, 2020). With so much at stake for both health and the economy, it is crucial to understand popular perceptions of the hazards of COVID-19 and the sources of their diversity. In this article, we ask: given mixed messages about the risk and impact of COVID-19, how do people perceive the threat to them, and the severity of its impact on others? Answers to this question are highly relevant to how marketing tools may be effectively deployed in support of broader public interests during and after the current pandemic.

This research builds on broader applications of the social, behavioral, and psychological sciences to understand and navigate the myriad challenges posed by COVID-19 (e.g., Kniffin et al., 2020; Van Bavel et al., 2020). Although the threat posed by the novel coronavirus was virtually unheard of outside the epidemiology community at the beginning of 2020, as the pandemic took hold, psychological research on the crisis began to rapidly appear. Consumer attitudes have been the subject of substantial early work. Campbell et al. (2020) proposed that the pandemic engenders feelings of “ontological insecurity,” accompanied by anxiety and a perceived loss of control, which increases reliance on familiar brands and activities. In keeping with this perspective, Galoni et al. (2020) found that contagious disease threats result in elevated fear and disgust, which is associated with a preference for familiar products. Conversely, Huang and Sengupta (2020) found that when contagious disease was made saliant, preference was reduced for typical products (e.g., orange juice) compared to atypical products (pomegranate juice), perhaps because typical objects are associated with greater numbers of people and higher contagion risk.

Without question, the threat of the pandemic has resulted in behavioral changes, mostly indicative of an increased desire for security, including reduced mobility (Malik et al., 2020; Yan et al., 2020). People have moved closer to the familiar and trusted novelty less. However, this has not translated to a general movement toward careful and reasonable information processing (Pennycock et al., 2020). Instead, information about the pandemic has been found to motivate elevated caution, including the intention to stockpile goods. Interestingly, these intentions were found to be reduced by contextualizing the pandemic with comparisons to other threats like traffic fatalities (Kim et al., 2020).

Collectively, this research indicates that a major challenge of the pandemic is psychological; ideally, threat perception is calibrated to motivate health-protecting behaviors without jeopardizing the broader functioning of an interdependent society. Some researchers have offered hopeful predictions (He and Harris, 2020) – adaptation to pandemic conditions may heighten both corporate social responsibility and ethical consumer choices, suggesting that interventions designed to facilitate prosociality during the pandemic and its aftermath may actually be enhanced in their effectiveness.

While the rapid proliferation of psychological research in response to COVID-19 is encouraging, our understanding of people's perceptions of the pandemic, and their associated behaviors, is incomplete. The current research advances that understanding by examining popular perceptions of the threat of COVID-19, as well as related prosocial vs. judgmental attitudes toward victims. We compare these attitudes with attitudes toward non-COVID-19 victims given the comparisons of COVID-19 with other sorts of adversities highlighted by high-profile government leaders and experts.

This research involves four studies with more than 4,100 participants within the United States in late April and early May of 2020 which examine how popular perceptions of COVID-19 compare to other adversities, including the flu and vehicle collisions. The outcome variables we investigate are willingness to help and perceptions of the risk of helping people and communities affected by COVID-19 (vs. non-COVID-19 threats), as well as perceptions of victims as contaminated, injured, and responsible for their condition.

We hypothesize that, on average, people perceive victims of COVID-19 as riskier to help, more responsible for their condition, more contaminated, and, are less willing to help them compared to non-COVID-19 victims (flu, car accident, HIV/AIDS, and severe storm). It is important to understand if people perceive COVID-19 victims in these ways, since these judgments have been shown to predict moral condemnation and blame (Alicke et al., 2015; Niemi and Young, 2016). If those affected by COVID-19 are viewed in ways that convey immorality, more so than victims of other adversities, then COVID-19 victims may be doubly victimized, by being subjected to unjust moral judgments in addition to the disease itself (Baldassarre et al., 2020).

Furthermore, we expect that there will be variability across individuals in perception of the threat of COVID-19 and its impact on victims, that is, whether the disease is injurious or contaminating. Some participants may focus on COVID-19 victims as suffering, whereas others may place less attention on their suffering compared to their capacity to endanger others by being infected. Our expectation is that this variability is explained by individual differences in people's moral values, as well as demographic characteristics including political orientation, gender, education, and income level.

According to Moral Foundations Theory (MFT; e.g., Haidt, 2007; Graham et al., 2009, 2011), conservative people tend to endorse the group-oriented “binding values” of (1) loyalty, (2) respect for authority, and (3) purity more highly than liberal people, who tend to favor the “individualizing values” that emphasize (4) fairness and (5) care. Unlike individualizing values which stipulate unbiased extension of moral concern, binding values foster group boundaries and “us vs. them” dynamics through (a) reciprocal bonds of loyalty, (b) deference to the authorities in the hierarchies that structure groups, and (c) commitment to preserving purity by rejecting people and behaviors that “contaminate” the integrity of the group. These features suggest that binding values might drive a heightened perception of the COVID-19 threat, despite the relationship of binding values with conservatism, and conservative rhetoric expressing skepticism about COVID-19 dangers.

We hypothesize that people higher in binding values may actually be *less* willing to help people and communities affected by COVID-19, given the rationale for expecting people high in binding values to tend to view those affected by COVID-19 as more contaminated and riskier to help. Furthermore, less willingness to help COVID-19 victims may be illustrative of a general tendency for people higher in binding values, regardless of politics, to judge victims with less sensitivity. Prior research shows that people higher in binding values are more likely to stigmatize victims as tainted and contaminated, judge victims as more responsible and blameworthy for their own victimization, and are less likely to defend victims of sexual harassment by confronting and reporting harassment (Niemi and Young, 2016; Goodwin et al., 2020; Niemi et al., 2020). By contrast, people higher in individualizing values, which are associated with increased sensitivity to suffering and do not emphasize contamination risks, have been found to be more prosocial (e.g., Graham et al., 2011; Iyer et al., 2012; Niemi and Young, 2013, 2016; Noser et al., 2015; Niemi et al., 2020) and may be more willing to help those affected by the coronavirus.

In addition to measuring attitudes and individual differences in values, we consider the contribution of political orientation, gender, education, and income level. We expect that conservative political orientation will be associated with negative attitudes toward both COVID-19 and non-COVID-19 victims based on previous links between binding values and victim blaming (Niemi and Young, 2016; Niemi et al., 2020), however, we expect binding values will account for relationships among conservativism and negative attitudes toward victims. We consider the role of gender, education, and income in attitudes since prior work suggests that there may be gender differences in risk-aversion (Byrnes et al., 1999; Hurley and Choudhary, 2020); and, education and income have been linked to COVID-19-related knowledge and behaviors (e.g., Irigoyen-Camacho et al., 2020). Finally, in order to illuminate the degree to which the salience of different moral values may be altered, and thereby influence attitudes, we examine whether increasing the salience of either binding or individualizing values through priming affects the outcome variables. Given prior research showing efficacy in priming moral values, we anticipated small effects in these studies for priming binding and individualizing values (Mooijman et al., 2018; Goenka and Thomas, 2020).

The studies were administered between April 24–27, 2020 and May 8–13, 2020 in the United States. During this time period, most Americans were under shut-down orders and there was increasing media focus on people's politically sourced disapproval of government COVID-19 policy, centered around coverage of protests against the shutdowns which began on April 15. In early May, shutdown orders largely remained, but some communities were already beginning to pursue re-opening.

## Study 1 Method

### Procedure

The Institutional Review Board at Cornell University approved all of the studies here (Protocol ID Number: 2004009568). All studies were preregistered through AsPredicted.org (see Supplementary Materials) as part of a series focused on understanding how MFT is related to attitudes concerning COVID-19. All studies were implemented using Qualtrics survey software and distributed to participants online *via* Prolific. De-identified data and study materials are archived in the corresponding author's online repository on OSF (see Supplementary Materials for link). Independent participant pools were recruited for each study.

The procedure for Study 1 involved (1) a moral values prime, (2) a vignette and series of questions measuring attitudes about individual victims, and (3) individual difference measures of moral values, political orientation, and demographics. In all studies, participants were excluded prior to analysis if they failed either of the two embedded attention checks, or an attention check at the end of the studies.

### Materials and Measures

Participants were first exposed to a moral values prime, used effectively in prior research (Mooijman et al., 2018; Goenka and Thomas, 2020). The primes described a warrior who exemplifies loyalty, respectfulness, and concern about purity (binding values), or caring and fairness (individualizing values), or who has good character (control).

Participants then read a vignette and were asked about their attitudes in a series of questions. In the vignette, participants read about “Dan,” who was affected by either COVID-19, the seasonal flu, or a car accident: “In March 2020, Dan drove across the country for work, and stopped at many cities and towns in several states. Along the way, he [contracted the coronavirus and became very sick; contracted the seasonal flu and became very sick; got into a serious car accident and sustained numerous injuries].” After the vignette, using Likert-scales (1–7), participants rated: “How responsible is Dan for the car accident?” (on one page); “How willing would you be to assist Dan?” and “How risky would it be for you to assist Dan?” (on the next page); and “How injured is Dan?” and “How contaminated is Dan?” (on the next page). We also examined but do not discuss here whether judgments would be affected by how contagiousness was conveyed (see Supplementary Materials for text of vignettes).

Next, we measured participants' endorsement of the five moral values of Moral Foundations Theory—caring, fairness, loyalty, obedience to authority, and purity values—with the Moral Foundations Questionnaire (MFQ-30, Graham et al., 2011). We averaged caring and fairness values for the individualizing values scores, and loyalty, authority, and purity values for the binding values scores.

Finally, participants took a brief demographics survey, and we measured political orientation with the item (Iyer et al., 2012): “When it comes to politics, do you usually think of yourself as liberal, moderate, conservative, or something else?”—a drop-down menu contained the choices: (1) Very liberal, (2) Liberal, (3) Slightly liberal, (4) Moderate/middle-of-the-road, (5) Slightly conservative, (6) Conservative, (7) Very conservative, (8) “Don't know/not political,” (9) “Libertarian,” (10) Other. We used selections 1–7 as a scale variable representing the extent of participants' self-identification as politically liberal or conservative.

## Study 1 Results

### Participants

Study 1 included 1,627 participants (836 female, 765 male, 26 other) with 72 exclusions based on failure of attention checks. The sample size was calculated to yield at least 50 participants per condition, plus 10 additional participants in each condition to account for typical rates of exclusion. The average age of the participants was 36.4 (*SD* = 13.0) years old; 90% of participants were not Hispanic or Latino, 10% were Hispanic or Latino, 74% White or European-American, 7% Black or African-American, 12% Asian or Asian-American, less than 1% Native American or Pacific Islander, 4% Multiracial, and 1.5% selected other. Combined annual income was: 22% < $30,000; 20% between 30,000 and 49,999; 18% between 50,000 and 69,999, 19% between 70,000 and 99,999, and 19% 100,000+. 43% of participants were liberal or very liberal; a similar percentage, 39%, was slightly liberal, middle-of-the-road, or slightly conservative; 12% were conservative or very conservative; 3% did not know or were not political; 1% selected libertarian; 1% selected other. Participants were from all four regions of the US: West (24%), Midwest (20%), Northeast (22%), and South (34%).

### Results

We conducted analyses of variance to investigate whether “affliction type:” COVID-19, seasonal flu, or car accident, affected whether participants considered the protagonist responsible, contaminated, and injured; as well as their willingness to help the protagonist, and, how risky they considered helping him. We also examined whether binding values increased perception of the victim as responsible, contaminated, and risky to help, and decreased willingness to help.

#### Responsibility

**Figure 1:**
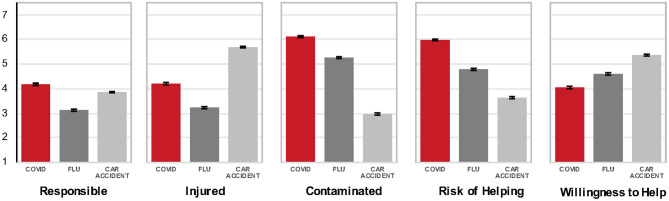
Mean ratings of responsibility, contamination, injury, risk of helping, and willingness to help a victim of COVID-19, the seasonal flu, or a car accident in Study 1 (conducted April 24–27, 2020) and Studies 2–3 (May 8–13, 2020; error bars indicate standard error of the mean).

We found a significant main effect of the prime [*F*_(2, 1546)_ = 3.41, *p* = 0.033, partial eta^2^ = 0.004], a significant main effect of affliction type [*F*_(2, 1546)_ = 71.20, *p* < 0.001, partial eta^2^ = 0.084], and no interaction. Ratings of Dan's responsibility (see means in Figure 1) were highest for being infected with COVID-19, higher than responsibility ratings for the car accident (*p* < 0.001) and seasonal flu (*p* = 0.003). Participants primed with binding values rated victim responsibility highest (*M* = 3.82, *SEM* = 0.07), significantly higher than participants primed with individualizing values (*M* = 3.58, *SEM* = 0.06, *p* = 0.009), but not significantly higher than control (*M* = 3.72, *SEM* = 0.07, *p* = 0.25).

#### Contamination

We found a significant main effect of the prime [*F*_(2, 1546)_ = 3.74, *p* = 0.024, partial eta^2^ = 0.005], a significant main effect of affliction type [*F*_(2, 1546)_ = 721.27, *p* < 0.001, partial eta^2^ = 0.482], and no interaction. Contamination ratings were highest in the case of COVID-19, higher than the flu (*p* < 0.001), and higher than the car accident (see means in Figure 1). Participants primed with binding values rated victim contamination higher (*M* = 4.74, *SEM* = 0.06) relative to individualizing values (*M* = 4.63, *SEM* = 0.06) which significantly differed from control (*M* = 4.87, *SEM* = 0.06; *p* = 0.006).

#### Injury, Riskiness, and Willingness to Help

The effect of affliction type was significant for injury, riskiness, and willingness to help. The car accident was considered most injurious, followed by COVID-19 (*p* < 0.001), and the flu [*p* < 0.001; *F*_(2, 1546)_ = 295.32, *p* < 0.001, partial eta^2^ = 0.276]. Helping Dan was rated riskiest when he contracted COVID-19, compared to the flu, or a car accident [*p* < 0.001; *F*_(2, 1546)_ = 354.65, *p* < 0.001, partial eta^2^ = 0.315]. Finally, participants were significantly less willing to help the COVID-19 victim compared to the flu victim and the car accident victim [*p* < 0.001; *F*_(2, 1546)_ = 85.78, *p* < 0.001, partial eta^2^ = 0.100).

#### Study 1 Summary

Results indicate that participants perceived the disease of COVID-19 to be more contaminating but less injurious than the flu or a car accident. Moreover, participants were less willing to help COVID-19 victims, who they considered more responsible for their affliction, and riskier to help. Finally, priming binding values had a small effect on attitudes, increasing ratings of victims, generally, as responsible and contaminated.

## Study 2 Method

### Procedure

In Study 2, the procedure was identical to Study 1.

### Materials and Measures

In Study 2, the materials and measures were identical to Study 1, with the exception that we also asked participants to identify a warrior quality from the vignette as an attention check.

## Study 2 Results

### Participants

Study 2 included 1,009 participants (510 female, 494 male, five other) with 73 exclusions based on failure of attention checks. The majority of participants in each prime condition identified the correct word from the vignette they read (individualizing 81%; binding 66%, control 71%). The sample size was calculated to yield at least 50 participants per condition, plus 10 additional participants to each condition to account typical rates of exclusion. The average age of the participants was 38.9 (SD = 13.7); 93% of participants were not Hispanic or Latino, 7% were Hispanic or Latino, 79% White or European-American, 7% Black or African-American, 10% Asian or Asian-American, <1% Native American or Pacific Islander, 3% Multiracial and 1% selected other. Combined annual income was: 18% < $30,000; 20% between 30,000 and 49,999; 18% between 50,000 and 69,999, 22% between 70,000 and 99,999, and 23% 100,000+. 42% were liberal or very liberal; a similar percentage, 41%, was slightly liberal, middle-of-the-road, or slightly conservative; 13% were conservative or very conservative; 2% did not know or were not political; ~2% selected libertarian; <1% selected other. Participants were from all four regions of the US: West (22%), Midwest (23%), Northeast (19%), and South (36%).

### Results

We conducted the same analyses as in Study 1, and replicated the effect of affliction type on all outcome variables (see Figure 1): *responsibility* [*F*_(2, 1000)_ = 52.22, *p* < 0.001, partial eta^2^ = 0.095], *contamination* [*F*_(2, 1000)_ = 570.43, *p* < 0.001, partial eta^2^ = 0.533], *injury* [*F*_(2, 1000)_ = 276.20, *p* < 0.001, partial eta^2^ = 0.356], *willingness to help* [*F*_(2, 1000)_ = 75.64, *p* < 0.001, partial eta^2^ = 0.131], and *risk* [*F*_(2, 1000)_ = 238.84, *p* < 0.001, partial eta^2^ = 0.323].

Once again, ratings of contamination and risk of helping were highest for COVID-19, followed by the flu and the car accident. Ratings of injury were again highest for the car accident, followed by COVID-19, and the flu. Ratings of responsibility were also highest in the case of COVID-19, followed by the car accident and the flu. Finally, willingness to help was highest for the car accident victim, followed by the flu, and COVID-19.

#### Study 2 Summary

The results of Study 2 replicate Study 1: relative to the flu and car accident victim, participants were again less willing to help the COVID-19 victim, who was considered riskier to help, more contaminated, less injured, and more responsible their affliction.

## Study 3 Method

### Procedure

The procedure used in Study 3 was identical to Study 2.

### Materials and Measures

Materials were identical to Study 2, with the exception that we attempted to replicate the priming results in Study 1 using a different set of moral values primes. We replaced the “warrior” primes from Studies 1–2 with a set of primes for binding and individualizing values that have been found to be effective in prior research (Mooijman et al., 2018). These primes involved reading a short passage about morality by a purported “morality scholar,” arguing that either the well-being of the group (binding values condition) or the well-being of individuals (individualizing values condition) is central to morality. Participants then wrote a brief response essay discussing their perspective on the scholar's ideas about morality. In the control condition, participants did not read or write a passage.

## Study 3 Results

### Participants

Study 3 included 1,026 participants (422 female, 593 male, 11 other) with 54 exclusions based on failure of attention checks. The sample size was calculated as in Study 2. The average age of the participants was 37.7 (SD = 14.7); 90% of participants were not Hispanic or Latino, 10% were Hispanic or Latino, 75% White or European-American, 10% Black or African-American, 8% Asian or Asian-American, 1% Native American or Pacific Islander, 4% Multiracial, and 2% selected other. Combined annual income was: 25% < $30,000; 20% between 30,000 and 49,999; 17% between 50,000 and 69,999, 20% between 70,000 and 99,999, and 19% 100,000+. 45% were liberal or very liberal; a similar percentage, 37%, was slightly liberal, middle-of-the-road, or slightly conservative; 12% were conservative or very conservative; 3% did not know or were not political; 1% selected libertarian; 2% other. Participants were from all four regions of the US: West (24%), Midwest (21%), Northeast (21%), and South (34%).

### Results

Analyses were identical to Studies 1–2: we again found significant main effects for affliction type on all of the outcome variables: *responsibility* [*F*_(2, 1016)_ = 34.87, *p* < 0.001, partial eta^2^ = 0.064], *contamination* [*F*_(2, 1016)_ =395.34, *p* < 0.001, partial eta^2^ = 0.438], *injury* [*F*_(2, 1016)_ = 277.91, *p* < 0.001, partial eta^2^ = 0.354], *willingness to help* [*F*_(2, 1016)_ = 47.22, *p* < 0.001, partial eta^2^ = 0.085], and *risk* [*F*_(2, 1016)_ = 219.19, *p* < 0.001, partial eta^2^ = 0.127].

#### Study 3 Summary

Replicating Studies 1–2, the results of Study 3 indicate (see Figure 1) that ratings of risk and contamination, but not injury, were highest for COVID-19, compared to the flu, and the car accident. Participants also again considered COVID-19 victims more responsible for their affliction and were least willing to help them.

## Studies 1–3 Additional Analysis

### Moral Values, Politics, Demographics, and Attitudes About COVID-19 Victims

Priming moral values produced results that partially supported our hypotheses: participants primed with binding values were more likely to consider victims responsible and contaminated. These findings are consistent with prior research on individual differences in moral values and attitudes about victims, which did not use priming.

In the present analyses, conducted on a merged dataset that combined the data from Studies 1–3, we investigated how participants' *surveyed* moral values (binding values, individualizing values) predicted attitudes about victims. We factored in politics, gender, education, and income, which have been linked to values and attitudes about risk and victims (i.e., politics 1–7: very liberal to very conservative, gender: male (0) and female (1), income in increments from 1-7: under $30–$100 K and over per year, and education from 1 to 6: some high school, high school, some university/college, university/college, graduate degree, doctoral, or professional degree (e.g., M.D., J.D., etc.).

We conducted a series of regression analyses on our outcome variables: (a) responsibility, (b) contamination, (c) injury, (d) perceived risk, and (e) willingness to help, for (1) COVID-19 victims and (2) non-COVID-19 (flu, car accident) victims. We entered moral values (binding values, individualizing values) in step one, and politics, education, gender, and income in step two. Full reporting of the 10 regression results is provided in the Supplementary Material. Table 1 presents the summary of each of the models. For each outcome variable—responsibility, willingness to help, injury, contamination, perceived risk—Model 1 represents the moral values predictors (individualizing and binding values). Model 2 represents the addition of politics and demographics to the model (i.e., education, income, gender, and politics). The *R*^2^ accounted for by each model, and *R*^2^ change values (Johnson and LeBreton, 2004) are presented in the first two columns, for each outcome variable.

**Table 1:** Results of regression analyses of judgments of responsibility, contamination, injury, riskiness of helping, and willingness to help for COVID-19 and non-COVID-19 victims in the combined dataset for Studies 1–3.

	**Responsibility**				**Willingness to Help**				**Injury**			
	***R*^**2**^**	***R*^**2**^ chng**.		**Sig. F change**	***R*^**2**^**	***R*^**2**^ chng**.		**Sig. F change**	***R*^**2**^**	***R*^**2**^ chng**.		**Sig. F change**
**COVID**												
Model 1	0.01	0.01	*F*_(2, 1134)_ = 3.65	***p*** **=** **0.026**	0.02	0.02	*F*_(2, 1134)_ = 8.67	***p*** **<** **0.001**	0.01	0.01	*F*_(2, 1134)_ = 7.63	***p*** **=** **0.001**
Model 2	0.02	0.01	*F*_(4, 1130)_ = 3.85	***p*** **=** **0.004**	0.03	0.02	*F*_(4, 1130)_ = 4.8	***p*** **=** **0.001**	0.03	0.01	*F*_(4, 1130)_ = 3.58	***p*** **=** **0.007**
**Non-COVID**												
Model 1	0.01	0.01	*F*_(2, 2221)_ = 13.09	***p*** **<** **0.001**	0.02	0.02	*F*_(2, 2221)_ = 26.93	***p*** **<** **0.001**	0.00	0.00	*F*_(2, 2221)_ = 3.85	***p*** **=** **0.021**
Model 2	0.02	0.00	*F*_(4, 2217)_ = 1.98	*p* = 0.095	0.03	0.00	*F*_(4, 2217)_ = 1.65	*p* = 0.160	0.01	0.00	*F*_(4, 2217)_ = 1.86	*p* = 0.114
	**Contamination**				**Risk**			
	***R***^**2**^	***R***^**2**^ **chng**.		**Sig. F change**	***R***^**2**^	***R***^**2**^ **chng**.		**Sig. F change**
**COVID**		
Model 1	0.02	0.02	*F*_(2, 1134)_ = 13.64	***p*** **<** **0.001**	0.02	0.02	*F*_(2, 1134)_ = 12.23	***p*** **<** **0.001**
Model 2	0.04	0.02	*F*_(4, 1130)_ = 5.28	***p*** **<** **0.001**	0.05	0.03	*F*_(4, 1130)_ = 7.61	***p*** **<** **0.001**
**Non-COVID**		
Model 1	0.02	0.02	*F*_(2, 2221)_ = 18.02	***p*** **<** **0.001**	0.02	0.02	*F*_(2, 2221)_ = 16.93	***p*** **<** **0.001**
Model 2	0.02	0.01	*F*_(4, 2217)_ = 3.68	***p*** **=** **0.005**	0.03	0.01	*F*_(4, 2217)_ = 5.92	***p*** **<** **0.001**

*Model 1 predictors: (Constant), Individualilzing, Binding*.

*Model 2 predictors: (Constant), Individualizing, Binding, Education, Gender, Income, Politics (liberal to conservative)*.

*All regressions used a two-step model with (Model 1) moral values (binding and individualizing values) entered in step one, and (Model 2) politics and demographics (education, gender, and income) entered in step two. For each model, R^2^ and R^2^ change are provided, along with the results and significance value for the F-test of the change in R^2^. Significance values less than 0.05 are bolded*.

The F statistic for the change in *R*^2^ values and associated significance levels (Table 1) indicate that for COVID-19 victims, moral values (Model 1) *and* the addition of politics and demographics (Model 2) significantly predicted all outcome variables.

However, for non-COVID-19 victims, moral values (Model 1) predicted all outcome variables, but politics and demographics (Model 2) did not. The addition of politics and demographics to moral values was significant only for ratings of contamination and risk (see Table 1). Thus, the results of the regression analyses indicate that moral values predict people's judgments of both COVID-19 and non-COVID-19 victims; however, politics and demographics consistently played a stronger role in judgments about COVID-19 victims.

## Study 4 Method

### Procedure

The procedure of Study 4 was similar to Studies 1–2 in that participants were primed, presented with a vignette, and surveyed about their attitudes and values.

### Materials and Measures

In Study 4, to shed light on whether moral values may affect judgments of groups differently than individuals, we investigated attitudes about communities rather than individual victims. Study 4 used the warrior prime from Studies 1–2, and participants read about an unnamed community: “Since March of 2020, residents of a large community have been hit hard by [an outbreak of the coronavirus; HIV/AIDS; a severe storm], and the city's infrastructure has been overwhelmed by victims needing care. Officials have called for donations, and volunteers to assist the relief effort in soup kitchens, homeless shelters, and medical facilities.” After the vignette, using Likert-scales (1–7), participants rated (each on a separate page): “How likely would you be to volunteer at [soup kitchen, homeless shelter, medical facilities]?” “How likely would you be to donate to [soup kitchen, homeless shelter, medical facilities]?” “How risky to your health do you think volunteering would be?” As in Studies 1–3, participants took a brief survey of demographic information, moral values and political orientation.

## Study 4 Results

### Participants

Study 4 included 571 participants (317 female, 218 male, four other) with 23 exclusions based on failure of attention checks. The sample size was calculated to yield at least 50 participants per condition, plus 10 additional participants in each condition to account for typical rates of exclusion. The average age of the participants was 35.7 (SD = 12.2); 92% of participants were not Hispanic or Latino, 8% were Hispanic or Latino, 79% White or European-American, 8% Black or African-American, 7% Asian or Asian-American, <1% Native American or Pacific Islander, 4% Multiracial and 1.3% selected other. Combined annual income was: 19% < $30,000; 20% between 30,000 and 49,999; 20% between 50,000 and 69,999, 21% between 70,000 and 99,999, and 19% 100,000+. 43% were liberal or very liberal; a similar percentage, 39% was slightly liberal, middle-of-the-road, or slightly conservative (39%); 13% were conservative or very conservative; 2% did not know or were not political; <1% selected libertarian, <1% selected other. Participants were from all four regions of the US: West (18%), Midwest (23%), Northeast (24%), and South (35%).

### Results

We used analyses of variance to investigate whether participants would be less willing to help (donate or volunteer) an unnamed community affected by COVID-19, and how they perceived the risk of helping. We were interested in potential differences in these outcome variables relative to HIV/AIDS, or a severe storm, as well as donation and volunteering targets: soup kitchen, homeless shelter, and medical facilities. In addition, we examined attitudes when binding values were made salient, vs. individualizing values or control (no prime): as in Studies 2–3, there was no effect of the moral values primes.

**Figure 2:**
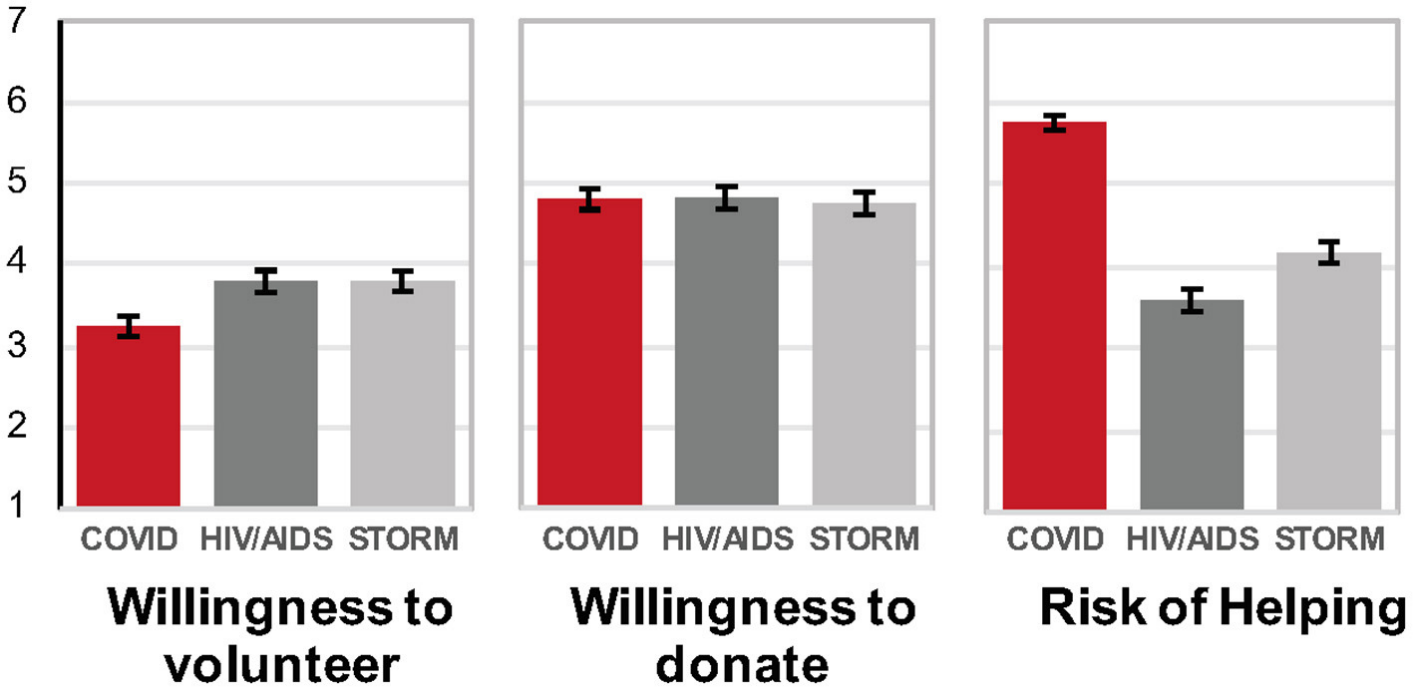
Average willingness to volunteer, willingness to donate, and perceived risk of helping in a community in Study 4 conducted April 24–27, 2020 (error bars indicate standard error of the mean).

Consistent with all previous studies, there was a significant main effect of affliction type for *volunteering* [*F*_(2, 508)_ = 6.58, *p* < 0.002, partial eta^2^ = 0.025]: participants were less willing to volunteer in the case of a community affected by COVID-19, compared to HIV/AIDS or a severe storm (*p'*s < 0.002; see Figure 2), as well as *riskiness* [*F*_(2, 508)_ = 86.18, *p* < 0.001, partial eta^2^ = 0.25]: people considered volunteering riskiest in a community affected by COVID-19, compared to HIV/AIDS or a severe storm (*p'*s < 0.001, see Figure 2). No effects were significant for *donation* amount. Finally, merging across volunteering and donating, there was a significant effect of helping location [*F*_(2, 1032)_ =53.32, *p* < 0.001, partial eta^2^ = 0.094]: people preferred to help a soup kitchen, followed by a homeless shelter, and medical facilities.

#### Study 4 Summary

Consistent with Studies 1–3 on individual victims, participants considered helping to be riskier, and were least willing to help a COVID-19-affected community, relative to a community affected by HIV/AIDS and storm victims.

## Discussion

The results of these studies, conducted in late April and early-mid May 2020 in the United States, illuminate how people perceive victims of COVID-19 compared to victims of other adversities. Participants were significantly less willing to help a person or community described as having been affected by COVID-19, compared to another kind of affliction (the flu; HIV/AIDS; car accident; severe storm).

Participants considered a person affected by COVID-19 to be more contaminated and responsible for their adverse circumstances, compared to a person with the flu or in a car accident. They did not, however, view the COVID-19 victim as more injured than a person in a car accident. Given the deadliness of the virus, it is striking that people perceived the threat of COVID-19 less in terms of its injuriousness than its contagion potential. Participants perceived the risk of helping a person or a community affected by COVID-19 to be significantly greater than the risk involved in helping those affected by more “familiar” adversities.

We found that moral values, demographics, and political commitments were related to these perceptions. Politics and values consistently predicted judgments of victims of COVID-19. However, values (and not politics or demographics) consistently predicted judgments for *non*-COVID-19 victims. The relationships between values and judgments of victims are consistent with previous research. Binding values have been found to predict increased perceptions of victims as blameworthy, responsible, and contaminated; individualizing values have been linked with prosociality (e.g., Iyer et al., 2012; Niemi and Young, 2013, 2016; Noser et al., 2015; Niemi et al., 2020). The finding that demographic factors and politics play a *greater* role in judgments of COVID-19 victims than non-COVID-19 victims indicates that participants' attitudes about COVID-19 may not be based on their stable moral principles (e.g., help those who are suffering). Instead, attitudes toward COVID-19 victims, but not other victims, are more likely informed by politics, likely in the form of messages from political figures or observations of what people with similar political views consider reasonable COVID-19 protocol.

Although we observed relationships between surveyed moral values and attitudes, we did not find that people's moral values or their responses to victims were reliably altered by our attempts to increase the salience of binding or individualizing values through priming. This may reflect the nature of our outcome variables, which were mainly pertinent to people's health and safety, rather than third-party moral judgments which have been influenced by values primes in previous work (Mooijman et al., 2018; Goenka and Thomas, 2020). The attitudes we measured may be more resistant than other-focused moral judgments to transient exogenous changes in the salience of particular moral values. Consistent with this possibility, the instance in which we observed a priming effect was in participants' third-party judgments of responsibility in Study 1, where priming binding values increased perceptions of victim responsibility.

To the extent that policymakers, charity organizations, and concerned individuals wish to persuade others to assist people affected by COVID-19, these results suggest it will be helpful to address people's safety concerns with education, while taking into account individual differences in politics and moral values. Future work should address which promotional tools, such as public-service messaging or manipulations of the salience of values, are conducive to helpful interventions in support of prosocial and health-related behaviors (e.g., Batson, 2011; Cameron and Payne, 2011; Waytz et al., 2013; Tannenbaum et al., 2015; Shariff et al., 2016; Amin et al., 2017; Benish-Weisman et al., 2019). Similarly, in light of research showing the varied impact of different kinds of thinking styles on the relevance of moral values (e.g., Li et al., 2017, 2018), it would be helpful if future research were to focus more closely on mechanisms that might moderate the relationships that politics and moral values have with perceptions of COVID-19. In light of prior research differentiating between liberals and conservatives on “economic” and “social” dimensions (e.g., Crawford et al., 2017), it remains to be understood how the pandemic fits into these categories, and whether attitudes about health constitute an independent third dimension for understanding how people tend to process the disease and its victims.

Some limitations of the present findings should be noted. The studies were cross-sectional using an online convenience sample, with self-report data. The limitations of cross-sectional data are well-known, and while some observers contend the difficulty is overstated (Spector, 2019), it is certainly manifest in this instance, because data was collected in a unique historical moment in the United States, April–May 2020, when the unprecedented social disruption of the pandemic was just beginning. It is likely that attitudes toward the dangers posed by the pandemic evolved over its course, and will continue to evolve over its aftermath; more studies at different points in time are needed. We note as well that while the quality of data obtained online may compare favorably to data obtained from other sources (Buhrmester et al., 2011; Hauser and Schwarz, 2016), the limitations of self-reports are well-known (Doris, 2002, 2015; Ross and Nisbett, 2011). Future research must supplement self-report data with objective behavioral measures; for example, the use of public transportation data to illuminate the pandemic's effect on people's mobility (Malik et al., 2020). Finally, our convenience sample, like all convenience samples raises the possibility of bias. Most notably, a majority of our participants were liberal or liberal leaning; consequently, given survey data indicating that Democrats overestimate the risks posed by COVID-9, while Republicans underestimate them (Rothwell and Desai, 2020), future research should more fully investigate the role of political orientation in COVID related attitudes and behavior. Nevertheless, we believe our research provides important insight into people's psychological processing of the pandemic.

The studies presented in this article help unpack the complex dynamics associated with how people and institutions are responding to COVID-19 and its victims. First, these results demonstrate clearly that people in the United States do *not* perceive those affected by COVID-19 like they do those with the flu. Prosocial inclinations toward COVID-19 victims are comparatively diminished. Second, people's differing moral values, politics, and demographic characteristics are associated with their reactions to victims of COVID-19; whereas reactions to *non*-COVID-19 victims are primarily predicted by moral values.

These findings should help make sense of the myriad downstream problems that have emerged as a result of COVID-19. For example, parents have faced difficult decisions about their children's education modality based on their own calculations of safety and risk. Additionally, public response to vaccine campaigns has not been uniformly enthusiastic, indeed, vaccine acceptability is subject to subtle framing effects (Kaplan and Milstein, 2021). A reasonable assessment of risk requires understanding whether the perceptions of the populace are accurate, and coming to terms with moralistic and politicized characterizations of COVID-19 sufferers (Baldassarre et al., 2020). Confronting counterproductive, stigmatizing characterizations of victims as contagion vectors, rather than disease sufferers, has the potential to improve the collective response to the COVID-19 pandemic.

## Data Availability Statement

The raw data supporting the conclusions of this article will be made available by the authors, without undue reservation. See the Supplementary Material for information about accessing the data via an online repository.

## Ethics Statement

The studies involving human participants were reviewed and approved by Institutional Review Board of Cornell University. The patients/participants provided their written informed consent to participate in this study.

## Author Contributions

LN analyzed the manuscript. All authors contributed to design of the studies, interpretation of the data, and writing of the manuscript.

## Conflict of Interest

The authors declare that the research was conducted in the absence of any commercial or financial relationships that could be construed as a potential conflict of interest.
