# Supplementary material for: It's Not the Flu: Popular Perceptions of the Impact of COVID-19 in the U.S
Source: Front Psychol. 2021 May 7;12:668518. doi: 10.3389/fpsyg.2021.668518 (PMC8138202; doi:10.3389/fpsyg.2021.668518)
Supplement: Supplementary file 1 [file Data_Sheet_1.pdf]

Supplementary Materials

In Study 1, we examined used three versions of the vignettes to investigate whether judgments of the individual would be affected by how contagiousness was conveyed. Based on Niemi, Leone, and Young (2021), we varied whether the target specifically (a) spread the disease, or (b) was affected completely, for a very long time.

(a) The following sentences were added to the vignette provided in the main text:

[COVID-19] Someone he socialized with on his trip also contracted the coronavirus [flu].

[Flu] Someone he socialized with on his trip also contracted the seasonal flu.

[Car accident] Another driver also sustained injuries in the accident.

(b) [All conditions] Dan was still in the hospital, and completely unwell, several weeks later.

Data and Materials

Data files, pre-registrations (AsPredicted.org), and study materials are uploaded to the corresponding author's OSF repository: https://osf.io/5p7jk/?view_only=3fe0a0f9fa954a258a1a82bbf1476ad5

Full Statistics for the Regression Models (Model summaries in Table 1)

Responsibility (Non-Covid)

Model		Unstandardized Coefficients		Standardized Coefficients			95% C I for B	
		B	SE	Beta	t	Sig.	Lower	Upper
1	*(Constant)*	3.264	.212		15.393	.000	2.848	3.680
	BINDING	.168	.034	.106	4.981	.000	.102	.234
	INDIVIDUALIZING	-.069	.042	-.035	-1.647	.100	-.152	.013
2	*(Constant)*	3.600	.266		13.535	.000	3.078	4.121
	BINDING	.206	.043	.129	4.822	.000	.122	.290
	INDIVIDUALIZING	-.087	.047	-.044	-1.827	.068	-.180	.006
	POLITICS	-.034	.023	-.042	-1.502	.133	-.079	.011
	GENDER	-.138	.059	-.049	-2.311	.021	-.254	-.021
	EDUCATION	-.018	.029	-.014	-.620	.535	-.076	.040
	INCOME	.002	.010	.003	.147	.883	-.019	.022

Responsibility (Covid)

Model		Unstandardized Coefficients		Standardized Coefficients			95% C I for B	
		B	SE	Beta	t	Sig.	Lower	Upper
1	*(Constant)*	3.223	.369		8.724	.000	2.498	3.948
	BINDING	.095	.056	.050	1.694	.091	-.015	.205
	INDIVIDUALIZING	.145	.071	.060	2.038	.042	.005	.285
2	*(Constant)*	4.051	.456		8.891	.000	3.157	4.945
	BINDING	.236	.069	.125	3.396	.001	.100	.372
	INDIVIDUALIZING	.045	.078	.019	.579	.563	-.108	.199
	POLITICS	-.133	.039	-.133	-3.447	.001	-.208	-.057
	GENDER	-.119	.104	-.034	-1.142	.254	-.324	.086
	EDUCATION	-.032	.051	-.019	-.632	.528	-.131	.067
	INCOME	-.024	.018	-.043	-1.375	.169	-.058	.010

Contamination (Non-Covid)

Model		Unstandardized Coefficients		Standardized Coefficients			95% C I for B	
		B	SE	Beta	t	Sig.	Lower	Upper
1	*(Constant)*	2.486	.283		8.773	.000	1.931	3.042
	BINDING	.176	.045	.082	3.893	.000	.087	.264
	INDIVIDUALIZING	.235	.056	.088	4.170	.000	.125	.346
2	*(Constant)*	2.446	.355		6.891	.000	1.750	3.142
	BINDING	.273	.057	.128	4.782	.000	.161	.384
	INDIVIDUALIZING	.141	.063	.053	2.229	.026	.017	.265
	POLITICS	-.082	.031	-.075	-2.684	.007	-.142	-.022
	GENDER	.183	.079	.049	2.302	.021	.027	.338
	EDUCATION	.042	.039	.024	1.077	.282	-.035	.120

	INCOME	-.007	.014	-.010	-.471	.638	-.034	.021

Contaminated (Covid)

Model		Unstandardized Coefficients		Standardized Coefficients			95% C I for B	
		B	SE	Beta	t	Sig.	Lower	Upper
1	*(Constant)*	4.841	.253		19.127	.000	4.344	5.337
	BINDING	.105	.038	.081	2.742	.006	.030	.180
	INDIVIDUALIZING	.211	.049	.127	4.336	.000	.116	.307
2	*(Constant)*	5.108	.311		16.407	.000	4.497	5.719
	BINDING	.184	.047	.141	3.871	.000	.091	.277
	INDIVIDUALIZING	.130	.053	.078	2.424	.016	.025	.235
	POLITICS	-.068	.026	-.098	-2.570	.010	-.119	-.016
	GENDER	.215	.071	.090	3.013	.003	.075	.355
	EDUCATION	-.080	.035	-.070	-2.315	.021	-.148	-.012
	INCOME	.006	.012	.015	.483	.629	-.018	.029

Injured (Non-Covid)

Model		Unstandardized Coefficients		Standardized Coefficients			95% C I for B	
		B	SE	Beta	t	Sig.	Lower	Upper
1	*(Constant)*	3.708	.281		13.206	.000	3.157	4.258
	BINDING	.062	.045	.029	1.386	.166	-.026	.150
	INDIVIDUALIZING	.126	.056	.048	2.258	.024	.017	.236
2	*(Constant)*	3.929	.352		11.156	.000	3.238	4.619
	BINDING	.129	.057	.062	2.286	.022	.018	.240
	INDIVIDUALIZING	.066	.063	.025	1.048	.295	-.057	.189
	POLITICS	-.057	.030	-.053	-1.870	.062	-.116	.003
	GENDER	.003	.079	.001	.037	.971	-.152	.157
	EDUCATION	.034	.039	.019	.880	.379	-.042	.111
	INCOME	-.026	.014	-.042	-1.894	.058	-.053	.001

Injured (Covid)

Model		Unstandardized Coefficients		Standardized Coefficients			95% C I for B	
		B	SE	Beta	t	Sig.	Lower	Upper
1	*(Constant)*	2.816	.367		7.666	.000	2.095	3.536
	BINDING	.114	.056	.061	2.054	.040	.005	.224
	INDIVIDUALIZING	.229	.071	.096	3.240	.001	.090	.368
2	*(Constant)*	2.610	.453		5.759	.000	1.721	3.499
	BINDING	.213	.069	.113	3.087	.002	.078	.349
	INDIVIDUALIZING	.161	.078	.067	2.069	.039	.008	.314
	POLITICS	-.090	.038	-.090	-2.352	.019	-.165	-.015
	GENDER	-.019	.104	-.006	-.186	.852	-.223	.184

	EDUCATION	.143	.050	.087	2.847	.004	.045	.242
	INCOME	-.011	.017	-.019	-.620	.535	-.045	.023

Risk (Non-Covid)

Model		Unstandardized Coefficients		Standardized Coefficients			95% C I for B	
		B	SE	Beta	t	Sig.	Lower	Upper
1	*(Constant)*	3.074	.250		12.290	.000	2.583	3.564
	BINDING	.206	.040	.110	5.179	.000	.128	.284
	INDIVIDUALIZING	.106	.050	.045	2.135	.033	.009	.204
2	*(Constant)*	3.008	.313		9.623	.000	2.395	3.621
	BINDING	.280	.050	.149	5.575	.000	.181	.378
	INDIVIDUALIZING	.017	.056	.007	.308	.758	-.092	.127
	POLITICS	-.062	.027	-.064	-2.281	.023	-.114	-.009
	GENDER	.271	.070	.082	3.876	.000	.134	.408
	EDUCATION	.023	.035	.015	.676	.499	-.045	.091
	INCOME	-.017	.012	-.030	-1.386	.166	-.041	.007

Risk (Covid)

Model		Unstandardized Coefficients		Standardized Coefficients			95% C I for B	
		B	SE	Beta	t	Sig.	Lower	Upper
1	*(Constant)*	4.823	.245		19.691	.000	4.343	5.304
	BINDING	.103	.037	.081	2.763	.006	.030	.175
	INDIVIDUALIZING	.188	.047	.117	3.993	.000	.096	.281
2	*(Constant)*	5.120	.300		17.060	.000	4.531	5.709
	BINDING	.176	.046	.139	3.837	.000	.086	.265
	INDIVIDUALIZING	.098	.052	.061	1.899	.058	-.003	.199
	POLITICS	-.060	.025	-.089	-2.347	.019	-.109	-.010
	GENDER	.271	.069	.117	3.938	.000	.136	.406
	EDUCATION	-.084	.033	-.077	-2.528	.012	-.150	-.019
	INCOME	-.008	.012	-.022	-.733	.464	-.031	.014

Help (Non-Covid)

Model		Unstandardized Coefficients		Standardized Coefficients			95% C I for B	
		B	SE	Beta	t	Sig.	Lower	Upper
1	*(Constant)*	3.414	.235		14.503	.000	2.953	3.876
	BINDING	.020	.037	.011	.527	.598	-.054	.093
	INDIVIDUALIZING	.339	.047	.152	7.233	.000	.247	.431
2	*(Constant)*	3.063	.295		10.370	.000	2.484	3.642
	BINDING	-.028	.047	-.016	-.587	.557	-.121	.065
	INDIVIDUALIZING	.384	.053	.173	7.280	.000	.280	.487
	POLITICS	.041	.025	.045	1.624	.104	-.009	.091

	GENDER	.008	.066	.002	.115	.908	-.122	.137
	EDUCATION	.018	.033	.012	.542	.588	-.046	.082
	INCOME	.020	.012	.037	1.707	.088	-.003	.043

Help (Covid)

Model		Unstandardized Coefficients		Standardized Coefficients			95% C I for B	
		B	SE	Beta	t	Sig.	Lower	Upper
1	*(Constant)*	3.083	.371		8.307	.000	2.355	3.811
	BINDING	-.091	.056	-.048	-1.615	.107	-.201	.020
	INDIVIDUALIZING	.279	.071	.115	3.898	.000	.138	.419
2	*(Constant)*	2.198	.457		4.811	.000	1.302	3.095
	BINDING	-.133	.070	-.070	-1.910	.056	-.270	.004
	INDIVIDUALIZING	.329	.078	.136	4.188	.000	.175	.483
	POLITICS	.038	.039	.038	.984	.325	-.038	.114
	GENDER	-.080	.105	-.023	-.761	.447	-.285	.126
	EDUCATION	.200	.051	.120	3.934	.000	.100	.300
	INCOME	.007	.018	.012	.382	.703	-.028	.041

REFERENCES

Niemi, L., Leone, C., and Young, L. (2021). Linguistic evidence for the dissociation between impurity and harm: differences in the duration and scope of contamination vs. injury. *Soc. Cogn.* 39:117. doi: 10.1521/soco.2021.39.1.117
